# Stratified assessment and warning regimen for prevention of acute adverse reactions to iodinated contrast media: results of 150,343 cases in a tertiary hospital

**DOI:** 10.1007/s11517-022-02751-5

**Published:** 2023-01-03

**Authors:** Heng Liu, Haiyan Qiu, Junling Liu, Lingru Wang, Li Zhao, Yaling Wang, Xue Li

**Affiliations:** 1 Department of Radiology, Daping Hospital, Army Medical University, No. 10 Changjiang Road, Yuzhong District, Chongqing, 400042 China; 2 Department of Radiology, PLA Rocket Force Characteristic Medical Center, No. 16 Xinjiekou Outer Street, Beijing, 100088 China; 3 Department of Nursing, Daping Hospital, Army Medical University, No. 10 Changjiang Road, Yuzhong District, Chongqing, 400042 China

**Keywords:** Risk management, Iodinated contrast media, Adverse drug reaction, Risk factors, Safety

## Abstract

For contrast-enhanced CT examinations, there is a lack of comprehensive optimal management strategies of risk factors to reduce the risk of iodinated contrast media (ICM)–induced acute adverse reactions (AAR). Here, we determine the relationship between the rate of ICM-AAR and a stratified assessment and warning (SAW) regimen, which integrated risk identification, stratification, early warning, and prevention. A total of 120,822 cases in the conventional assessment period (58 years ± 15, 55.25% men) and 150,343 cases (58 years ± 14, 55.83% men) in the SAW period were enrolled. The results showed that the total AAR incidence in the SAW period (414/150,343, 0.28%) was lower than that in the conventional assessment period (506/120,822, 0.42%, *P* < 0.001), in which the proportion of AAR patients decreased by one-third. It mainly presented as decreases in mild and moderate reactions (*P* < 0.001), and a decrease in the proportion of moderate AAR patients (*P* = 0.001). Subgroup analysis showed lower mild and moderate AAR incidence in patients with different risk levels and with different ICM injection parameters following SAW regimen. We concluded that SAW regimen was associated with lower incidence of mild and moderate AAR, and decreased proportion of moderate AAR patients, which held potential for improved ICM safety.

**Graphical abstract:**

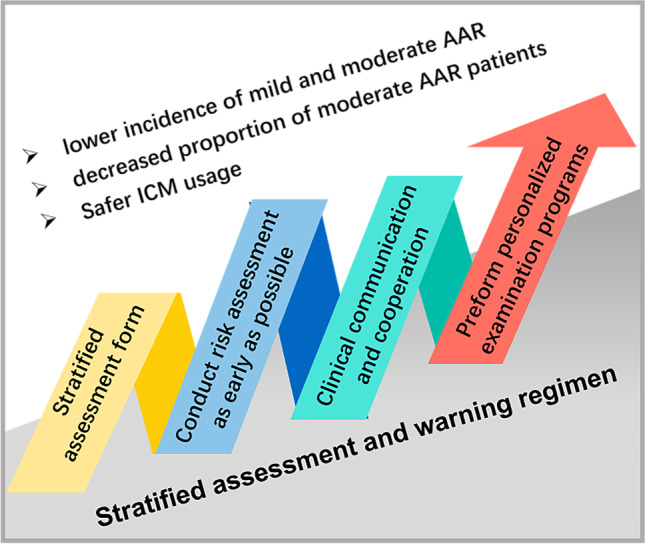

**Supplementary information:**

The online version contains supplementary material available at 10.1007/s11517-022-02751-5.

## Introduction

Iodinated contrast media (ICM)–assisted contrast-enhanced CT (CECT) examinations are widely and daily conducted for diagnostic procedures in imaging departments worldwide. Although patients can benefit from their usage, ICM possess inherent risk to cause acute adverse reactions (AAR) with different severities [1, 2]. AAR is defined as an adverse reaction that occurs within 1 h post-ICM injection, which is not related to the purpose of ICM administration under normal usage and dosage. The incidence of AAR ranges from 0.34 to 0.73%, accounting for 96.3% of the total adverse reactions, and AAR can be serious and even life-threatening [3–6]. The occurrence of AAR follows sporadic and unpredictable patterns, and is likely related to the complicated and combined effects of miscellaneous risk factors [4, 7, 8]. Current prophylactic approaches are mainly focused on preventing the recurrent AAR in patients with a previous ICM-AAR history, such as corticosteroids and/or antihistamines premedication, intradermal skin test, and change ICM from the culprit formulation [9–14]. For the whole population, several prophylactic approaches have also been reported, such as lower dose and injection speed, extrinsic warming of ICM to 37℃, and stratified dietary preparation regimen [15–18]. However, there is a lack of solid high-quality evidence of their effectiveness [19–21]. Up to now, the efficacy of comprehensive prevention strategies involving the optimal management of ICM-AAR risk factors in the whole population has not been systematically evaluated.

Timely and accurate risk factor assessment can screen out high-risk patients in advance, which is of clinical importance to reduce the frequency of ICM-AAR, and to ensure maximum safety of ICM usage. To prevent possible AAR, current ICM usage guidelines proposed several common risk factors that deserved special attention and highlighted the importance of risk assessment [1, 2]. In the vast majority of Chinese medical institutions, initial risk assessment is usually performed by clinicians when ordering examinations. Due to the limitations of expertise field and knowledge background, clinicians have very limited awareness of ICM-AAR risk factors, which may lead to misleading assessment results. The risk re-assessment is usually performed by radiology nurses in the form of simple question-and-answer (yes or no) on-site just before examination, according to the risk factors that deserve special attention outlined in ICM usage guidelines [1, 2]. Inquiring about some complicated risk factors may be not detailed or in-depth enough attributed to a tight schedule on-site, making the accurate recognition and stratified management of risk factors difficult. The lack of objectively and quantitatively standardized assessment programs may lead to inconsistent assessment results among different medical staff and medical institutions. Furthermore, some patients with advanced age and poor self-knowledge ability are unable to communicate accurately and describe their medical information completely. The inaccurate estimation of risk factors may instigate subsequent excessive/insufficient prevention measures, which inevitably leads to a series of clinical issues [22, 23]. On the other hand, if high-risk circumstances were recognized when inquiry, no adequate and appropriate preparation could be implemented due to time constraints [24, 25]. In this scenario, such a patient population had to be rescheduled for elective examinations, giving rise to delayed radiology diagnosis time and a waste of medical personnel resources [25–28]. It is urgently demanded to establish a scientific, practicable, and widely applicable comprehensive optimal management strategy for ICM-AAR risk factors.

From April 2017, our hospital undertook a quality improvement project that involved a stratified assessment and warning (SAW) regimen for ICM-AAR risk factors, which integrated risk identification, stratification, early warning, and prevention, and aimed to standardize the management of ICM-AAR risk factors. With the radiology nurses as the main body, through structured cooperation of the radiological team as well as their collaboration with clinical departments, stratified assessment and personalized management of risk factors were implemented during the period from patient appointment to ICM injection. After the conclusion of our quality improvement project, we realized that the SAW regimen also provided an opportunity to create clinical evidence on whether the SAW regimen has any effect on the risk of AAR. This retrospective study aimed to determine the relationship between the SAW regimen and ICM-AAR occurrence.

## Materials and methods

### Study participants

**Fig. 1:**
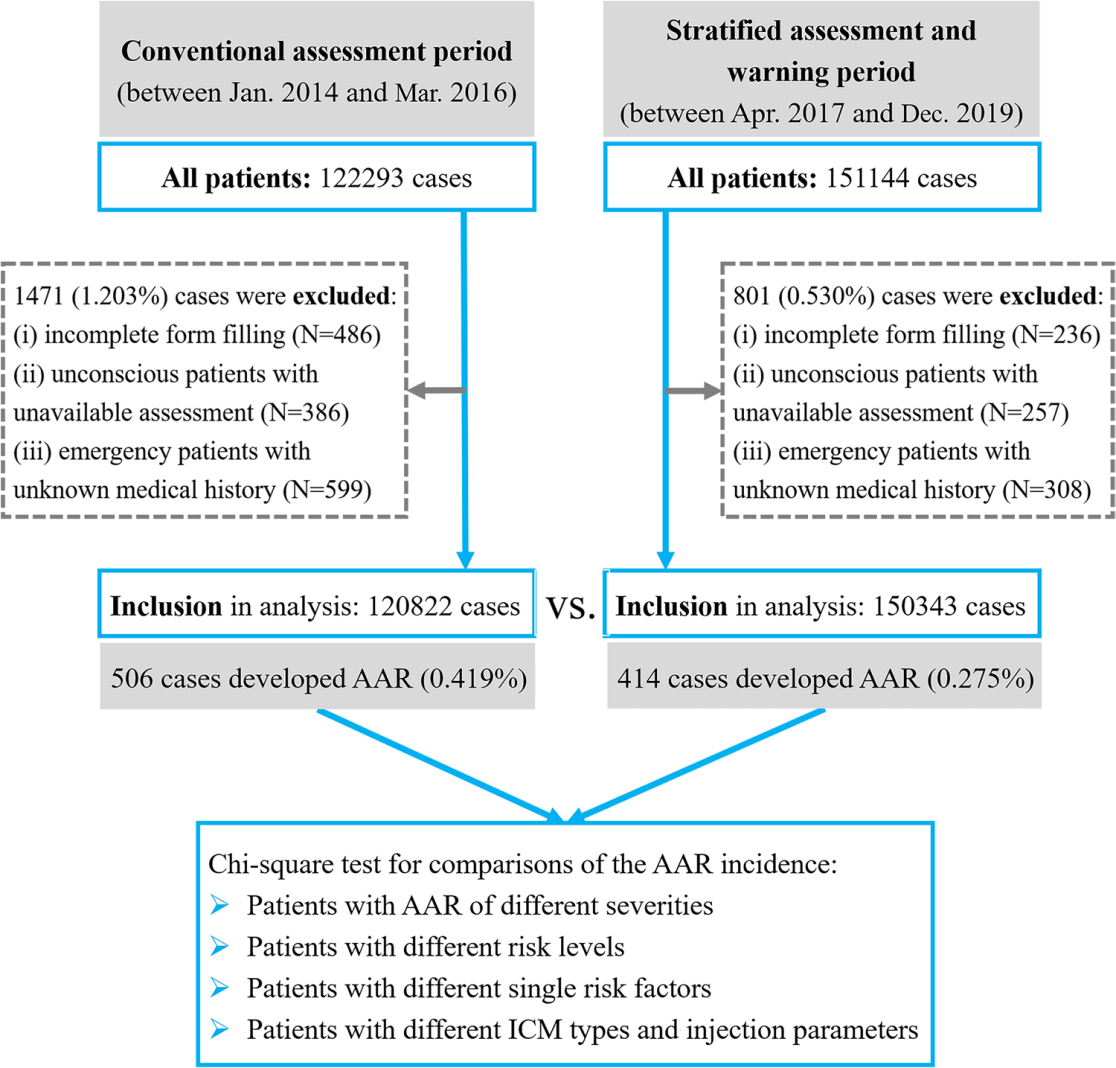
Study flow diagram

CECT scan data were collected and retrospectively analyzed from our hospital, a tertiary general medical institution with 2600 beds that provides all medical and surgical services. This study was approved by the institutional review board of our hospital. The written informed consent was exempted because of the retrospective nature of this study, which would not affect the rights of the participants, and all personal data were removed and coded as arbitrary numbers. The research flow chart is shown in Fig. 1. Inclusion criteria: (i) patients who met the indications and underwent routine CECT from January 2014 to March 2016 (conventional assessment period) and from April 2017 to December 2019 (SAW period) [1, 2]; (ii) patients who had risk factors outlined in international ICM usage guidelines [1, 2], but needed CECT for disease diagnosis; (iii) there was no age limit for patients. Exclusion criteria: (i) patients with incomplete form data filling; (ii) unconscious patients with unavailable assessment; (iii) emergency patients with unknown medical history. We allowed a transition period of 12 months (from April 2016 to March 2017) for the quality improvement project to fully permeate the examinations, and data from during this period were not analyzed in this study.

### CECT imaging equipment and ICM used

Philips Brilliance iCT Scanner (Royal Dutch Philips Electronics Ltd, Amsterdam, The Netherlands) and GE LightSpeed VCT® (GE Healthcare, Milwaukee, WI, USA) were used for CECT examinations. Non-ionic ICM were intravenously injected by a high-pressure injector (Ulrich Medical® Inc., Ulm, Germany). The injection doses and injection rates of ICM were adopted according to our institutional protocol [16]. The ICM used included Iodixanol 270 (GE Healthcare, London, UK), Ioversol 320 (Jiangsu Hengrui Medicine Co., Ltd, Jiangsu, China), Iodixanol 320 (Jiangsu Hengrui Medicine Co., Jiangsu, China), Iohexol 350 (Yangtze River Pharmaceutical Co., Ltd, Jiangsu, China), Iopamidol 350 (Bracco, Milan, Italy), Iobitridol 350 (Guerbet, Paris, France), and Iopromide 370 (Bayer Healthcare, Leverkusen, Germany).

### Assessment and management of ICM-AAR risk factors in the conventional assessment group

Initial risk assessment was performed by clinicians at the time of ordering examinations. The risk re-assessment was performed on-site just before examination by radiology nurses in the form of simple question-and-answer (yes or no), according to the risk factors that deserve special attention outlined in international ICM guidelines [1, 2]. The radiology nurses with over 8 years of work experience asked to fill out the conventional assessment form (Supplementary Table 1), informed about the risks of ICM injection, and asked the patients to sign the informed consent form for ICM injection. The patients were closely observed during and after examinations, and abnormal reactions were treated in time and routine hydration was performed. For patients with risk factors [1, 2], the radiology nurses reported to the radiologists, and the radiologists should communicate with the clinicians about the individualized risk–benefit ratio of examination, countermand the examination directly, reschedule for elective examinations after clinical treatment if necessary, or consider alternative imaging modalities with comparable diagnostic values.

### Stratified assessment and management of ICM-AAR risk factors in the SAW group

In the SAW period, a whole-process comprehensive management integrating risk identification, stratification, early warning, and prevention was performed according to different risk levels (Fig. 1). A self-design stratified assessment form was used for risk assessment (Supplementary Table 2). The risk factors were classified into different risk levels, including high risk, low risk, and no risk (including unknown risk). According to different risk stratification, corresponding comprehensive intervention was implemented (Supplementary Table 3), which was mainly consisting of clinical communication, patient communication, and full predictive intervention process. The re-assessment process prior to examination was moved forward as early as possible post-appointment. Adverse drug reactions (ADR) record cards and risk warning signboards were used as warning tools for accurate identification and risk stratification labelling, respectively. For more details, please see the supplemental materials. Detail comparisons of conventional assessment and SAW regimen are shown in Supplementary Table 4.

### Data documentation and quality control

All patients who underwent CECT examination routinely filled out the conventional assessment form or the updated stratified assessment form for risk factors (Supplementary Table 2), and all patients who developed ADR filled out the uniform ADR record form (Supplementary Table 5). AAR were observed and recorded by radiology nurses, and their severities (mild, moderate, severe) were determined according to ACR Manual on Contrast Media (Version 10.3) [1]. For details on quality control, please see the supplemental materials.

### Statistical analysis

All variables were descriptively analyzed. Continuous variables were described in terms of mean values and standard deviation. The counting data was presented in terms of frequencies and percentages (%). A chi-square test was performed for rate comparison on SPSS 22.0 (IBM, Chicago, USA), and *P* < 0.05 was considered statistically significant. The rate differences and 95% confidence interval (CI) were estimated using the VassarStats website http://vassarstats.net/index.html.

## Results

### Study participants

A total of 273,437 cases underwent CECT examinations, in which “case” was equal to the number of ICM administration. A total of 2272 cases were excluded (Fig. 1), including 722 cases with incomplete form data filling, 643 unconscious cases with unavailable assessment, and 907 emergency cases with unknown medical history. After exclusion, the complete analysis consisted of data from 120,822 eligible cases in the conventional assessment group (58 years ± 15, 66,573 men [55.25%]) and 150,343 eligible cases (58 years ± 14, 83,937 men [55.83%]) in the SAW group (Table 1).

**Table 1:** Summary of patient characteristics

	Characteristics	Conventional assessment group (%)	SAW group (%)
Number of patients		120,822	150,343
Gender	Male	66,753 (55.25)	83,937 (55.83)
	Female	54,069 (44.75)	66,406 (44.17)
Age (years)	Age range (years)	0–104	0–102
	Mean age (years)	58 ± 15	58 ± 14
	0–29	5013 (4.15)	5592 (3.72)
	30–69	89,741(74.28)	111,922 (74.44)
	≥ 70	26,068 (21.58)	32,829 (21.84)
Examination region	Coronary CTA	23,718 (17.71)	26,869 (16.62)
	Head and neck CTA/CTP	40,836 (30.49)	40,741 (25.20)
	Other regions	69,392 (51.81)	94,039 (58.18)
Type of ICM	LOCM	102,208 (84.59)	132,906 (88.40)
	IOCM	18,614 (15.41)	17,437 (11.60)
Injection dose	< 100 mL	104,971 (86.88)	138,702 (92.26)
	≥ 100 mL	12,264 (10.15)	11,596 (7.71)
	Unknown	3587 (2.97)	45 (0.03)
Injection speed	< 5 mL/s	70,835 (58.63)	97,967 (65.16)
	≥ 5 mL/s	42,878 (35.49)	52,332 (34.81)
	Unknown	7109 (5.88)	44 (0.03)

*ICM*, iodinated contrast media; *SAW*, stratified assessment and warning; *CTA*, CT angiography; *CTP*, CT perfusion; *LOCM*, low-osmolality contrast media; *IOCM*, iso-osmolality contrast media

More than one examination region might be involved in one CECT examination

### The relationship between SAW regimen and AAR occurrence

**Fig. 2:**
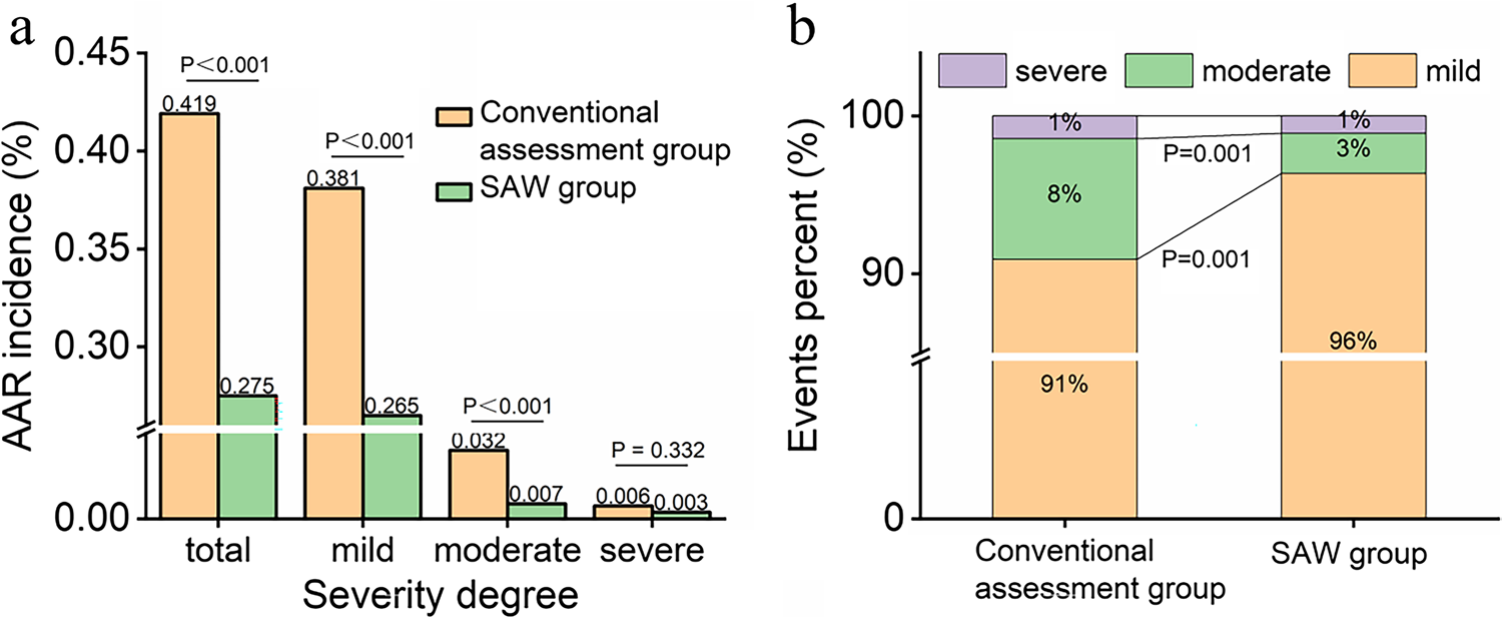
Comparison of the incidence and proportions of AAR with different severities in the conventional assessment group and SAW group

A comparison of the incidence and proportions of AAR with different severities is shown in Fig. 2. The total AAR incidence in the SAW group (414 of 150,343 examinations, 0.28%) was lower than that in the conventional assessment group (506 of 120,822 examinations, 0.42%, *P* < 0.001), that is to say, the proportion of patients who developed AAR decreased by about one-third. The decrease in AAR occurrence mainly presented as decreases in mild (*P* < 0.001) and moderate reactions (*P* < 0.001, Fig. 2a), and a decrease in the proportion of moderate AAR patients (*P* = 0.001, Fig. 2b). No statistical difference was found in the severe AAR incidence (*P* = 0.33).

**Fig. 3:**
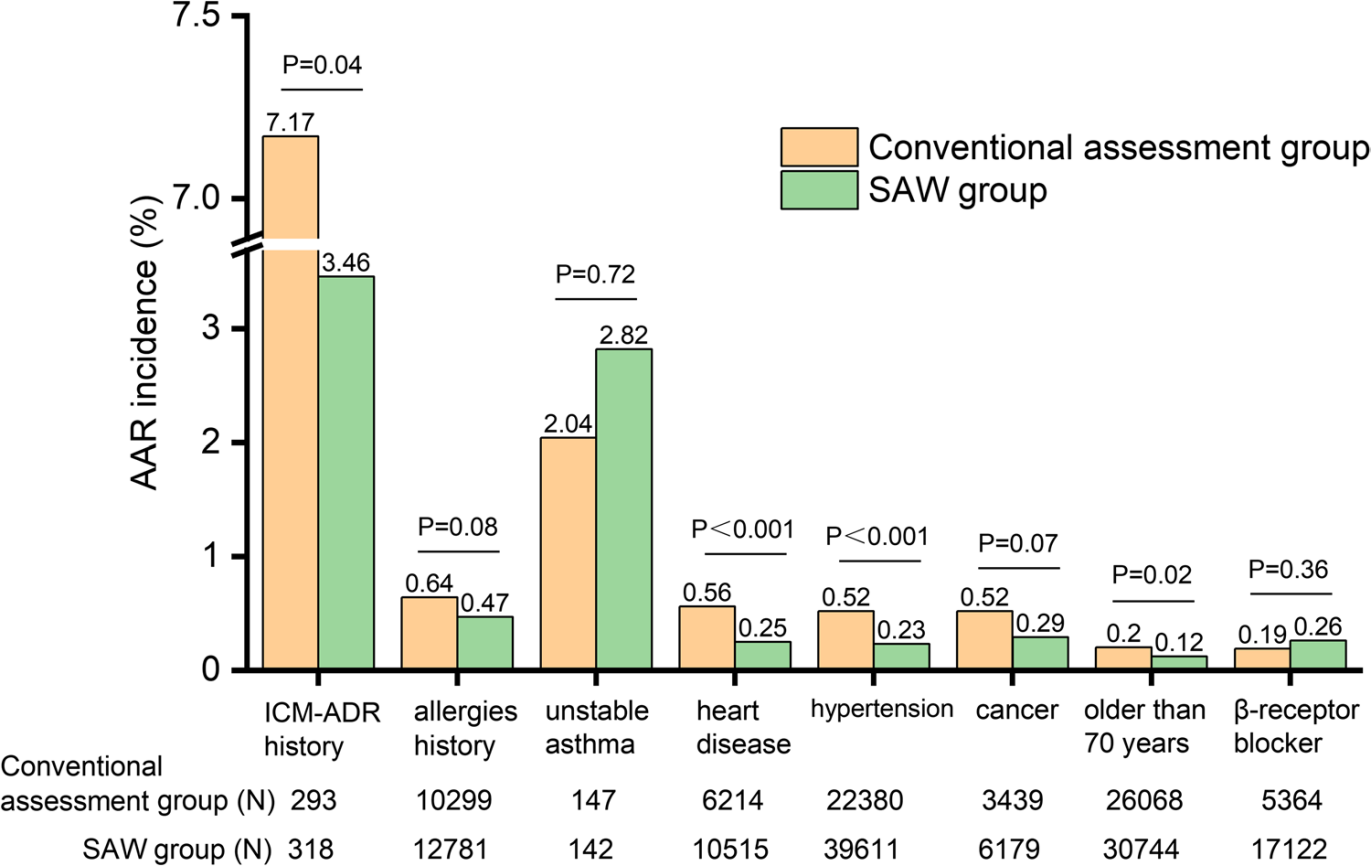
Comparison of the AAR incidence in patients with different single-risk factors in the conventional assessment group and SAW group

The AAR incidence in patients with risk factors is shown in Table 2. Whether patients with no risk or at risk, low risk or high risk, single-risk factor or multi-risk factor, the SAW group exhibited lower AAR incidence than that in the conventional assessment group (*P* < 0.05). The rate difference in high-risk patients (0.52%) was greater than that in low-risk patients (0.12%), and the proportion of AAR in high-risk patients was reduced by about 58%. A comparison of the AAR incidence in patients with different single risk factors is shown in Fig. 3. The AAR incidence in patients with ICM-AAR history, heart disease, hypertension, and advanced age (≥ 70 years) in the SAW group was lower than that in the conventional assessment group, respectively (*P* < 0.05). The proportion of these populations who developed AAR was reduced by about 52%, 60%, 56%, and 41%, respectively. Forty patients who developed AAR in the conventional assessment period and subsequently received ICM again during the SAW period were analyzed (data not shown). The ADR history records showed that another non-culprit ICM was used for every patient and no prophylactic medication was performed. Interestingly, none of them developed recurrent events.

**Table 2:** The AAR incidence in patients with risk factors

		Conventional assessment group (%)	SAW group (%)	*P* value	RD (95%CI)
With/without risk factors	No risk	0.38 (372/97,439)	0.25 (274/108,786)	< 0.001	0.13 (0.08, 0.18)
	At risk	0.57 (134/23,383)	0.34 (140/41,557)	< 0.001	0.24 (0.13, 0.35)
	*P* value	< 0.001	0.01		
Patients with no risk	Mild AAR	0.35 (339/97,439)	0.24 (264/108,786)	< 0.001	0.105 (0.06, 0.15)
	Moderate AAR	0.03 (29/97,439)	0.007 (8/108,786)	< 0.001	0.02 (0.01, 0.03)
	Severe AAR	0.004 (4/97,439)	0.002 (2/108,786)	0.43	0.002 (− 0.003, 0.007)
Patients at risk	Mild AAR	0.52 (121/23,383)	0.33 (135/41,557)	< 0.001	0.19 (0.09, 0.30)
	Moderate AAR	0.04 (10/23,383)	0.007 (3/41,557)	0.01	0.04 (0.01, 0.07)
	Severe AAR	0.01 (3/23,383)	0.005 (2/41,557)	0.36	0.008 (− 0.01, 0.03)
Risk level	Low risk	0.45 (75/16,746)	0.32 (99/30,583)	0.03	0.12 (0.005, 0.24)
	High risk	0.89 (59/6637)	0.37 (41/10,974)	< 0.001	0.52 (0.26, 0.77)
	*P* value	< 0.001	0.44		
Number of risk factors	Single risk	0.55 (116/21,074)	0.32 (117/36,135)	< 0.001	0.23 (0.11, 0.34)
	Multi-risk	0.78 (18/2309)	0.42 (23/5422)	0.049	0.36 (− 0.04, 0.75)

*AAR*, acute adverse reactions; *SAW*, stratified assessment and warning; *RD*, rate difference; *CI*, confidence interval

The AAR incidence in patients with different ICM injection parameters is shown in Table 3. Whether iso-osmolality contrast media (IOCM) or low-osmolality contrast media (LOCM), the SAW group exhibited lower AAR incidence than that in the conventional assessment group (*P* = 0.01, < 0.001, respectively), which was mainly reflected in mild and moderate AAR. Whether low or high injection dosages and speeds, the AAR incidence in the SAW group was lower than that in the conventional assessment group (*P* < 0.05).

**Table 3:** The AAR incidence in patients with different ICM injection parameters

		Conventional assessment group (%)	SAW group (%)	*P* value	RD (95% CI)
Types of ICM	IOCM	0.69 (129/18,614)	0.47 (82/17,437)	0.01	0.22 (0.07, 0.38)
	LOCM	0.37 (377/102,208)	0.25 (332/132,906)	< 0.001	0.12 (0.07, 0.17)
	*P* value	< 0.001	< 0.001		
IOCM	Mild AAR	0.63 (117/18,614)	0.46 (80/17,437)	0.03	0.17 (0.02, 0.32)
	Moderate AAR	0.06 (11/18,614)	0.005 (1/17,437)	0.01	0.05 (0.02, 0.10)
	Severe AAR	0.005 (1/18,614)	0.005 (1/17,437)	1.00	0 (− 0.03, 0.03)
LOCM	Mild AAR	0.34 (343/102,208)	0.24 (319/132,906)	< 0.001	0.10 (0.05, 0.14)
	Moderate AAR	0.03 (28/102,208)	0.008 (10/132,906)	< 0.001	0.02 (0.01, 0.03)
	Severe AAR	0.006 (6/102,208)	0.002 (3/132,906)	0.29	0.004 (0, 0.01)
Injection dose	< 100 mL	0.39 (413/104,971)	0.28 (385/138,702)	< 0.001	0.12 (0.07, 0.16)
	≥ 100 mL	0.60 (73/12,264)	0.25 (29/11,596)	< 0.001	0.35 (0.18, 0.52)
	*P* value	0.01	0.59		
Injection speed	< 5 mL/s	0.34 (238/70,835)	0.27 (262/97,967)	0.01	0.07 (0.02, 0.12)
	≥ 5 mL/s	0.57 (245/42,878)	0.29 (152/52,332)	< 0.001	0.28 (0.20, 0.37)
	*P* value	< 0.001	0.42		

*ICM*, iodinated contrast media; *AAR*, acute adverse reactions; *SAW*, stratified assessment and warning; *RD*, rate difference; *CI*, confidence interval; *LOCM*, low-osmolality contrast media; *IOCM*, iso-osmolality contrast media

## Discussion

Current guidelines lack large-scale data for optimal management of ICM-AAR risk factors and effective prevention of ICM-AAR [1, 2]. Based on a quality improvement project, our study evaluated the efficacy of comprehensive optimal management of risk factors in reducing the risk of ICM-AAR in the whole population for the first time (Table 4). The whole-process SAW regimen implemented in a large clinical cohort (150,343 cases), built a comprehensive risk management process, and realized timely identification and effective management of risk factors. The results showed that the total AAR incidence in the SAW period was lower than that in the conventional assessment period. The decrease in AAR occurrence mainly presented as decreases in mild and moderate reactions, and a decrease in the proportion of moderate AAR patients. The results indicate that the SAW regimen holds great potential for improved ICM safety.

**Table 4:** Detailed comparison between our study and related works of literature involving prophylactic strategies to reduce the rate of adverse reactions to ICM

Author year [ref.]	Prophylactic strategy	Research type	Study objects	ICM used	Adverse reactions observed	Incidence of adverse reactions	Main findings
The present study	Stratified assessment and warning regimen	Retrospective	All the patients	Iodixanol 270, Ioversol 320, Iodixanol 320, Iohexol 350, Iopamidol 350, Iobitridol 350, Iopromide 370	AAR	(i) Conventional assessment period: 0.42% (506/120,822); (ii) Stratified assessment and warning period: 0.28% (414/150,343)	Stratified assessment and warning regimen was associated with lower incidence of mild and moderate AAR, and decreased proportion of moderate AAR patients
Park et al. 2019 [15]	Lower dose and injection speed	Retrospective	Age ≥ 18 years	Iohexol, Iopamidol, Ioversol, Iomeprol, Iobitridol, Iodixanol	Acute HSR	(i) Control period: 1.86% (468/25,119); (ii) Intervention period: 1.42% (376/26,491)	Reduction in the dose and injection speed of ICM was associated with a lower incidence rate of acute HSR
Liu et al. 2022 [16]	Stratified dietary preparation regimen	Retrospective	Non-emergency adult patients	Iodixanol 270, Ioversol 320, Iodixanol 320, Iohexol 350, Iopamidol 350, Iobitridol 350, Iopromide 370	Adverse drug reactions	(i) Fasting group: 0.211% (105/49,676); (ii) Non-fasting group: 0.254% (197/77,524)	Unrestricted food ingestion would not increase the overall risk of adverse reactions
Lee et al. 2020 [17]	Intradermal skin test (IDT)	Prospective	Age ≥ 18 years	Iohexol, Iopamidol, Ioversol, Iomeprol, Iobitridol, Iodixanol, Iopromide	HSR	(i) Positive IDT result: 0/15 (ii) Negative IDT result: 19/2828	Routine IDT has no clinical value for prediction of HSR
Zhang et al. 2018 [18]	Extrinsic warming of ICM to 37℃	Retrospective	All the patients	Iopromide 370, Iopamidol 370, Iohexol 350, Iopromide 300	Allergic-like reactions	(i) No warming: 0.32% for Iopromide 370, 0.14% for Iopamidol 370, 0.32% for Iohexol 350 (ii) Warming: 0.21% for Iopromide 370, 0.10% for Iopamidol 370, 0.13% for Iohexol 350	Extrinsic warming to 37 °C was associated with a lower rate of allergic-like reactions
Sohn et al. 2020 [9]	IDT; ICM substitution	Retrospective	Patients with previous HSR	Iomeprol, Ioversol, Iohexol, Iopamidol, Iobitridol, Iopromide	Recurrent HSR	For patients with previous severe HSR: (i) Change ICM without the common side chain: 24.0%; (ii) Change ICM with the common side chain: 7.8%	For patients with previous severe HSR, ICM substitution without a common side chain or a negative skin test result is safer
Kwon et al. 2019 [10]	IDT	Prospective	Patients with previous immediate HSR	Iomeprol, Ioversol, Iodixanol, Iohexol, Iopamidol, Iobitridol, Iopromide	Recurrent HSR	(i) Positive IDT results and exposed to an IDT-positive ICM: 4/5; (ii) Positive IDT results and exposed to an IDT-negative ICM: 0/11; (iii) Negative IDT results and exposed to an IDT-positive ICM: 0/5; (iv) Negative IDT results and exposed to an IDT-negative ICM: 2/17	When the culprit ICM showed IDT-positive, IDT-negative ICM could be selected as a safer alternative. When the culprit ICM was IDT-negative, further IDT had no value in predicting recurrent HSR
Lee et al. 2017 [11]	Stratified premedication	Retrospective	Patients with previous immediate HSR	Iobitridol, Iohexol, Iomeprol, Iopamidol, Iopromide	Breakthrough reaction	For cases with a severe index reaction: (i) Single dose of corticosteroid: 55.6%; (ii) Double doses of corticosteroid: 17.4%	Severity-tailored stratified premedication could reduce the risk of breakthrough reactions
McDonald et al. 2021 [12]	ICM substitution; steroid premedication	Retrospective	Patients with a previous allergic-like reaction	Iohexol, Iopromide, Iodixanol	Repeat allergic-like reactions	(i) Same ICM and steroid premedication:19% (80/423); (ii) Different ICM and no steroid premedication: 3% (10/322); (iii) Different ICM and steroid premedication: 3% (5/166)	Compared with steroid premedication and the culprit ICM, ICM substitution was more effective for preventing repeat allergic-like reactions
Park et al. 2018 [13]	Premedication; ICM substitution	Retrospective	Patients with mild immediate HSR	Iobitridol, Iohexol, Iomeprol, Iopamidol, Iopromide, and Ioversol	Recurrent HSR	(i) Control: 31.1% (85/273); (ii) Changing the culprit agent only: 12% (105/872); (iii) Combination of changing the ICM and antihistamine premedication: 7.6% (148/1947)	A combination of changing the culprit ICM and antihistamine premedication provided the best outcome for preventing recurrent HSR
Abe et al. 2016 [14]	Premedication; ICM substitution	Retrospective	Patients with previous adverse reactions	Iopamidol 300, Iopamidol 370, Iohexol 300	Repeat adverse reactions	(i) Control: 27.7% (61/220); (ii) Premedication alone: 17.3% (47/271); (iii) Changing ICM alone: 5.2% (3/58); (iv) Premedication and changing ICM: 2.7% (6/222)	Premedication could be protective against repeat adverse reactions, and ICM substitution was more effective

*AAR*, acute adverse reactions; *ICM*, iodinated contrast media; *HSR*, hypersensitivity reactions; *IDT*, intradermal skin test

### The relationship between SAW regimen and reduced AAR occurrence

Given the critical role of ICM in modern medical imaging and the huge population for CECT examinations worldwide, a substantial reduction in the potential risks of ICM administration is of great significance to alleviating the social, medical, and economic burden [24, 25]. The decreased AAR occurrence in the SAW period mainly presented as decreased incidence of mild and moderate AAR, and a decreased proportion of moderate AAR patients. This is of great importance for alleviating the rescue and disposal loads of medical personnel. To investigate the relationship between the SAW regimen and patient subgroups, patients were classified into no-risk and at-risk based on the assessment results. The greater rate difference of AAR in at-risk patients suggested that the SAW regimen had a more direct and remarkable effect on this population. The effect of the SAW scheme on patients with no risk and at risk was mainly manifested as decreases in mild and moderate AAR. Further analysis revealed that the AAR incidence in patients with whether high risk or low risk, single-risk factor or multi-risk factor in the SAW period was lower than the conventional assessment period, respectively. The greater rate difference in high-risk patients suggested that the SAW regimen had a more direct and remarkable effect on this population.

For the AAR occurrence in patients with various single-risk factors, patients with ICM-ADR history, heart disease, hypertension, and advanced age (≥ 70 years) in the SAW group had lower AAR incidence than that in the conventional assessment group respectively. The proportion of these populations who developed AAR was reduced by about 52%, 60%, 56%, and 41%, respectively. As the possible interference caused by the different basic physical conditions among different patients was eliminated, the aforementioned self-control study results from 40 patients with ICM-ADR history strongly indicated that the SAW program was associated with prevention and control of AAR reoccurrence. While having no statistical difference, the AAR incidence in patients with history of other allergies or cancer was lower. This indicated that some treatment measures (e.g., replacing the culprit ICM, controlling the injection dosages and speeds) might have a certain effect on inhibiting AAR occurrence, which was consistent with previous reports [3, 9, 11, 14].

This effect of ICM injection dosages and speeds on AAR occurrence in the conventional assessment group was consistent with reports in the literature [29, 30]. Interestingly, there was no statistically significant difference in the AAR incidence in patients with high and low injection dosages in the SAW group, but both were lower than those with low injection dosages in the conventional assessment group. This might suggest that following the SAW regimen, the AAR incidence in patients with high dosages could be reduced to a level similar to or below that of patients with low dosages in the conventional assessment group. The injection speed data showed similar results. These results further indicated that the SAW regimen had a certain directive significance for selecting appropriate ICM parameters. It suggested that if conditions permit in clinical practice, LOCM could be chosen for replacement for high-risk patients with an IOCM-AAR history, and avoid high injection dose and injection speed whenever possible.

### Possible explanations for lower AAR occurrence following SAW regimen

As aforementioned, there was a close relationship between the SAW regimen and decreased AAR occurrence. The re-assessment process prior to examination was moved forward as early as possible post-appointment by radiology nurses in the SAW period to guarantee appropriate and adequate preparation of patients prior to examination. Our intervention regimen was not limited to anti-allergy pretreatment for patients with ICM-ADR histories; it emphasized non-pharmacological intervention prior to examination. The purpose was to develop specific treatment plans for patients who needed specific treatments, mainly including adequate fluid intake and treatment measures against high-risk underlying diseases. Furthermore, ADR history record cards and risk warning signboards were introduced. Clear and exact ADR documentation could help nurses obtain an appropriate and adequate medical history for the patients, and quickly find out the culprit ICM responsible for previous ADR, so that another non-culprit ICM could be recommended in subsequent examination procedures, which is usually tolerated very well by the patients [14, 31]. The risk warning signs reminded technicians to pay close attention to the patient’s condition, and control the injection dosage and speed of ICM for at-risk patients. It also enabled nurses to select appropriate and individual nursing measures according to different risk levels, implement dynamic monitoring during the examination, and observe closely after examination. Taken together, AAR could be timely recognized and treated, and the severe AAR incidence could be minimized in patients at risk. Considering advantages including unified standards, standardized procedures, simple methods, and specific treatment measures, the SAW regimen possessed high promotion value.

This study has some limitations. First, this study was not a randomized trial and the confounders were not adjusted when making comparisons, which might exist unrecognized changes and unmeasured differences in patient populations. Prospective multi-center randomized controlled trials will help to further validate the clinical efficacy of the SAW regimen. Second, the phenomena that abandoned examinations directly, rescheduled for elective examinations, and selected alternative imaging modalities arising from risk overestimation were significantly reduced in the SAW period. However, the exact number of these cases was not documented in detail. Furthermore, we focused on the AAR occurrence in patients with different risk levels, but did not further address the relationship between different severities of underlying risk diseases and AAR. Nevertheless, our results showed that following a standardized SAW regimen, at-risk patients, especially high-risk patients and the ones with multiple risk factors, had a significantly reduced AAR incidence in real-life practice. The cumulative effect of multiple risk factors deserves further verification.

## Conclusion

As a comprehensive optimal management process of risk factors, the SAW regimen implemented in a large clinical cohort was associated with lower incidence of mild and moderate AAR, and alleviated AAR severity, which held potential for improved ICM safety.

## Supplementary information

Below is the link to the electronic supplementary material.

Supplementary file1 (DOCX 56 KB)

## Glossary

*ICM*: Iodinated contrast media
*AAR*: Acute adverse reactions
*ADR*: Adverse drug reactions
*SAW*: Stratified assessment and warning
*CI*: Confidence interval
*LOCM*: Low-osmolality contrast media
*IOCM*: Iso-osmolality contrast media

## References

[1] American College of Radiology Committee on Drugs and Contrast Media (2018) ACR manual on contrast media. Available at https://www.acr.org/Clinical-Resources/Contrast-Manual. Accessed 31 Dec 2022

[2] European Society of Urogenital Radiology (2018) ESUR Guidelines on Contrast Agents. Available at http://www.esur.org/fileadmin/content/2019/ESUR_Guidelines_10.0_Final_Version.pdf. Accessed 31 Dec 2022

[3] Cha MJ, Kang DY, Lee W (2019). Hypersensitivity reactions to iodinated contrast media: a multicenter study of 196 081 patients. Radiology.

[4] Li X, Liu H, Zhao L (2017). Clinical observation of adverse drug reactions to non-ionic iodinated contrast media in population with underlying diseases and risk factors. Br J Radiol.

[5] Li X, Chen J, Zhang L (2015). Clinical observation of the adverse drug reactions caused by non-ionic iodinated contrast media: results from 109,255 cases who underwent enhanced CT examination in Chongqing, China. Br J Radiol.

[6] Yi KM, Li X (2022). Fatal noncardiogenic pulmonary edema related to nonionic, iso-osmolar iodine contrast medium: one case report. BMC Pulm Med.

[7] Wang L, Qiu H, Chen L et al (2022) Hemodynamic effects of intravenous bolus injection of iopromide 370 twice in abdominal contrast-enhanced CT and coronary CTA dual-site sequential examinations. Med Biol Eng Comput. 10.1007/s11517-022-02705-x 10.1007/s11517-022-02705-x 36342597

[8] Li X, Liu H, Zhao L (2018). The effect of preparative solid food status on the occurrence of nausea, vomiting and aspiration symptoms in enhanced CT examination: prospective observational study. Br J Radiol.

[9] Sohn KH, Seo JH, Kang DY, Lee SY, Kang HR (2021). Finding the optimal alternative for immediate hypersensitivity to low-osmolar iodinated contrast. Invest Radiol.

[10] Kwon OY, Lee JH, Park SY (2019). Novel strategy for the prevention of recurrent hypersensitivity reactions to radiocontrast media based on skin testing. J Allergy Clin Immunol Pract.

[11] Lee SY, Yang MS, Choi YH (2017). Stratified premedication strategy for the prevention of contrast media hypersensitivity in high-risk patients. Ann Allergy Asthma Immunol.

[12] McDonald JS, Larson NB, Kolbe AB (2021). Prevention of allergic-like reactions at repeat CT: steroid pretreatment versus contrast material substitution. Radiology.

[13] Park SJ, Kang DY, Sohn KH (2018). Immediate mild reactions to CT with iodinated contrast media: strategy of contrast media readministration without corticosteroids. Radiology.

[14] Abe S, Fukuda H, Tobe K, Ibukuro K (2016). Protective effect against repeat adverse reactions to iodinated contrast medium: premedication vs. changing the contrast medium. Eur Radiol.

[15] Park HJ, Son JH, Kim TB (2019). Relationship between lower dose and injection speed of iodinated contrast material for CT and acute hypersensitivity reactions: an observational study. Radiology.

[16] Liu H, Zhao L, Liu J (2022). Change the preprocedural fasting policy for contrast-enhanced CT: results of 127,200 cases. Insights Imaging.

[17] Lee JH, Kwon OY, Park SY (2020). Validation of the prescreening intradermal skin test for predicting hypersensitivity to iodinated contrast media: a prospective study with ICM challenge. J Allergy Clin Immunol Pract.

[18] Zhang B, Liu J, Dong Y (2018). Extrinsic warming of low-osmolality iodinated contrast media to 37°C reduced the rate of allergic-like reaction. Allergy Asthma Proc.

[19] Hsieh C, Wu SC, Kosik RO, Huang YC, Chan WP (2022) Pharmacological prevention of hypersensitivity reactions caused by iodinated contrast media: a systematic review and meta-analysis. Diagnostics (Basel) 12:1673 10.3390/diagnostics12071673 PMC9320945 35885578

[20] Umakoshi H, Nihashi T, Shimamoto H (2020). Pharmacologic and non-pharmacologic interventions to prevent hypersensitivity reactions of non-ionic iodinated contrast media: a systematic review protocol. BMJ Open.

[21] Umakoshi H, Nihashi T, Takada A (2022). Iodinated contrast media substitution to prevent recurrent hypersensitivity reactions: a systematic review and meta-analysis. Radiology.

[22] Ananthakrishnan L, Parrott DT, Mielke N, Xi Y, Davenport MS (2021). Fidelity of electronic documentation for reactions prompting premedication to iodinated contrast media. J Am Coll Radiol.

[23] Böhm I, Morelli J, Nairz K, Silva Hasembank Keller P, Heverhagen JT (2017). Myths and misconceptions concerning contrast media-induced anaphylaxis: a narrative review. Postgrad Med.

[24] Meucci E, Radice A, Fassio F (2020). Diagnostic approach to hypersensitivity reactions to iodinated contrast media: a single-center experience on 98 patients. Eur Ann Allergy Clin Immunol.

[25] Lakhal K, Ehrmann S, Robert-Edan V (2020). Iodinated contrast medium: is there a re(n)al problem? A clinical vignette-based review. Crit Care.

[26] Böhm I, Nairz K, Morelli JN, Keller PS, Heverhagen JT (2017). Iodinated contrast media and the alleged “iodine allergy”: an inexact diagnosis leading to inferior radiologic management and adverse drug reactions. Rofo.

[27] Davenport MS, Perazella MA (2020) Use of intravenous iodinated contrast media in patients with kidney disease: consensus statements from the American College of Radiology and the National Kidney Foundation. Radiology 294:660–668 10.1148/radiol.2019192094 31961246

[28] Wulf NR, Schmitz J, Choi A, Kapusnik-Uner J (2021). Iodine allergy: common misperceptions. Am J Health Syst Pharm.

[29] Park HJ, Son JH (2019) Relationship between lower dose and injection speed of iodinated contrast material for CT and acute hypersensitivity reactions: an observational study. Radiology 293:565–572 10.1148/radiol.2019190829 31617789

[30] Böhm IB (2020). Lower dose and lower injection speed of iodinated contrast media: a new strategy to reduce the incidence rate of immediate hypersensitivity reactions. Quant Imaging Med Surg.

[31] Böhm IB, van der Molen AJ (2020). Recommendations for standardized documentation of contrast medium-induced hypersensitivity. J Am Coll Radiol.

