# Supplementary material for: Stratified assessment and warning regimen for prevention of acute adverse reactions to iodinated contrast media: results of 150,343 cases in a tertiary hospital
Source: Med Biol Eng Comput. 2023 Jan 3;61(3):709–20. doi: 10.1007/s11517-022-02751-5 (PMC9918562; doi:10.1007/s11517-022-02751-5)
Supplement: Supplementary file 1 — Supplementary file1 (DOCX 56 KB) [file 11517_2022_2751_MOESM1_ESM.docx]

**Supplemental Material**

**Stratified assessment and warning regimen** **for prevention of acute adverse reactions to iodinated contrast media: results of 150343 cases in a tertiary hospital**

*Heng Liu^1, 2#^, MD,* *Haiyan Qiu^1 #^, MM,* *Junling Liu^1#^, MB, Lingru Wang^1^, MM,* *Li Zhao^1^, MB, Yaling Wang^3*^, MM,* *Xue Li^1^**^*^, MB*

^1^Department of Radiology, Daping Hospital, Army Medical University, No. 10 Changjiang Road, Yuzhong District, Chongqing 400042, China.

^2^Department of Radiology, PLA Rocket Force Characteristic Medical Center, No. 16 Xinjiekou Outer Street, Beijing 100088, China.

^3^Department of Nursing, Daping Hospital, Army Medical University, No. 10 Changjiang Road, Yuzhong District, Chongqing 400042, China.

**^#^**H.L., H.Y.Q. and J.L.L. contributed equally to this work.

***^*^*Correspondence to**: Y.L.W. (E-mail: 930706229@qq.com; Tel: +86-23-68757401); X.L. (E-mail: lixue928136@163.com; Tel: +86-23-687746905; Fax: +86-23-687746900).

**Materials and Methods**

**Stratified assessment of risk factors in the SAW group**

Design stratified assessment form: Continuous quality improvement was carried out on the basis of the conventional assessment. The assessment items for risk factors were optimized and updated, and the degrees of risk factors were stratified. (i) Determine the risk assessment items: Through our previous observation results of more than 400000 CECT patients [1-4], we found that patients with asthma, heart disease, hypertension, ICM-ADR history, other allergy history, tumor chemotherapy, and elder patients presented a higher ADR incidence. To ensure the rationality and effectiveness of the screening process, the items in the assessment form were improved by combining our institutional large-scale results and international ICM guidelines [5; 6]. (ii) Determine the contents in the assessment form: An expert seminar was held by five radiologists, five radiology technicians, and five radiology nurses. All of them were senior members of the local Contrast Media Safety Committee with deputy senior professional titles and above, and had worked in tertiary hospitals for more than 8 years. The risk factors were discussed item-by-item and were classified into different risk levels, including high risk, low risk, and no risk (including unknown risk). Ultimately, the screening items were determined in the stratified assessment form (**Supplementary Table 3**).

**Pre-control strategies in the SAW group**

Move forward re-assessment process: The re-assessment process prior to examination was moved forward as early as possible post-appointment. The radiology nurses with over 8 years of work experience filled out the stratified assessment form item-by-item. For patients with unclear information descriptions, their medical histories and related test results were obtained through the medical information system. The assessment of diseases like thyroid diseases, hypertension, heart insufficiency, renal insufficiency, and asthma were based on their final clinical diagnosis.

ADR record cards and risk warning signboards: (i) ADR record card: The card recorded the date and time for previous ADR, culprit ICM brands, injection dosages and speeds, clinical symptoms, severities, treatment and outcomes. The card could accurately reflect the events that occurred, and was held by the patients themselves and brought to any other hospital for check-up before examination. (ii) Risk warning label: The risk degrees were divided into high-risk, low-risk and no-risk, and the high-risk and low-risk was colored in red and yellow respectively. For patients with multiple risk factors, the most serious risk factor was adopted as the basis for determining the risk degree. The patients’ risk levels were carefully determined by one doctor-in-charge and one nurse-in-charge both with over 8 years of work experience. If disagreements occurred, they discussed with clinicians to determine the final assessment results.

**Determine** **intervention strategies in the SAW group**

According to different risk stratification, corresponding comprehensive intervention was implemented (**Supplementary Table 4**): (i) Clinical communication: For high-risk patients, the radiology nurses reported to the radiologists, and the radiologists fully communicated with the clinicians to evaluate the risk-benefit ratio for examination and determined whether the patients would receive ICM. For patients with histories of severe ICM-ADR, unstable asthma, and severe cardiovascular diseases, preventive treatments were carried out by clinicians and the examination time was reasonably rescheduled. For patients with histories of moderate to severe ICM-ADR, antihistamine and corticosteriods were used for prophylactic therapy according to ICM guidelines. For patients with histories of unstable asthma, and severe cardiovascular diseases, symptomatic treatment was performed to stable the condition of patients. (ii) Patient communication: The radiology nurses informed the patients about the assessment decisions and explained the precautions during examination. Ordinary patients signed a general informed consent form. Patients with risk factors signed an informed consent form with caution, which emphasized more on the possible adverse consequences post examinations for high-risk patients. (iii) Full predictive process: For patients with risk factors but need examination for disease diagnosis, the radiology nurses first evaluated the efficacy of pretreatment after the patients arrived at the radiology department during the post-appointment period, and prepared contingency plans for different risk factors. Different risk signs were labeled on the checklists to remind technicians of formulating personalized examination programs, including adopting lower injection dosages and rates of ICM, switching to another class of ICM for patients with ICM-ADR histories, performing dynamic electrocardiograph monitoring during the examination, closely observing the condition changes, asking whether the patients had abnormal reactions. High-risk patients were followed up after examination by dedicated staff. If any abnormality occurred, the emergency department or residents were informed to immediately start the rescue emergency plan, and follow up was well-performed.

**Data documentation**

The ADR record form mainly included basic information of patients, risk factors, examination sites, ICM names, injection dosages and injection speeds. For patients who developed AAR, the dates of occurrence, time of occurrence, severities, ICM names, clinical manifestations, vital signs, treatment measures, drugs used, remission time, and clinical outcomes were recorded.

**Quality control**

To ensure the accuracy and consistency of the assessment results, the radiologists, technicians, and nurses received formal trainings in advance, such as thematic lectures, on-site demonstrations, and situational presentations. The training contents include ICM-related risk factors and intervention methods, assessment form usages, clinical information acquirements, informed consents, and how to implement emergency treatments. The evaluation was conducted by dedicated staff, and the data was collected with unified standards and checked daily, and the missing items were completed in time. After data registration, two medical staff with over 8 years of work experience checked the original data blind-to-blind to ensure the data accuracy and completeness.

**Supplementary Table 1.** ICM-ADR Risk Conventional Assessment Form

| Name: Age: Gender: Male □ Female □ Body weight: ID number: | | | |
| --- | --- | --- | --- |
| Patient Source: Outpatient □ Emergency □ Inpatient □ Health Examination □ | | | |
| Previous ICM administration | 1. Yes □  2. No □  3. Unknown □ | Previous ICM reactions | 1.Yes □  2.No □  3.Unkown □ |
| History of other allergies | 1. Yes □  Specific allergy □ Asthma □ Pollinosis □  Drugs □ Foods □ Others:  2. No □  3. Unknown □ | | |
| Other risk factors | 1. Yes □  Impaired renal function □ Prior kidney surgery □  Diabetes require treatment □  Hypertension □ Coronary heart disease □ Asthma □  Cardiac insufficiency □ Hyperthyroidism □  Gout □ Age > 70 □ Medication with renal toxicity □  2. No □ | | |
| Underlying diseases | 1. Cardiac □ 2. Renal □ 3. Diabetes □  4. Respiratory system □ 5. Hepatic/gall □ 6. Pelvic □  7. Gastrointestinal □ 8. Nervous system □ 9. Hematological □  10. Thyroid □ 11. Unknown □ 12. Hypertension □  13. Chemoradiotherapy □ 14. Others: | | |
|  |  |  |  |

**Supplementary Table 2**. ICM-ADR Risk Stratified Assessment Form

| Name: Age: Sex: Male □ Female □ Body weight: ID number: | | | | | |
| --- | --- | --- | --- | --- | --- |
| Patient Source: Outpatient □ Emergency □ Inpatient □ Health Examination □ | | | | | |
| Reaction type | | | Risk degree | | |
|  |  |  | High risk | Low risk | No risk |
| Immediate reaction | Type A reaction | Severe cardiac disease or heart insufficiency (grade Ⅲ-Ⅳ) | Symptomatic | - | Good medication control |
|  |  | Hypertension (grade Ⅱ-Ⅲ) | Symptomatic | - | Good medication control |
|  | Type B reaction | Previous history of ICM-ADR | Yes | - | - |
|  |  | History of other atopy requiring treatment | - | Yes | - |
|  |  | History of unstable asthma | Symptomatic | - | Good medication control |
| Delayed reaction | Type A reaction | PC-AKI risk  (renal insufficiency)  eGFR (mL/min/1.73m^2^) | <30 | 30-59 | ≥60 |
|  | Type B reaction | History of delayed hypersensitivity | - | Yes | - |
| Very late reaction | Type A reaction | Thyroid disease | Acute thyrotoxicosis | 1. Untreated Graves’ disease  2. Multinodular goiter  3. Thyroid autonomy is being treated | - |
| Uncertain | | β-blockers | - | Yes | - |
|  |  | Tumor (under chemotherapy) | - | Yes | - |
|  |  | Advanced age | - | - | Yes |
|  |  | Others | - | - | Yes |

**Abbreviations:** ICM, iodinated contrast media; ADR, adverse drug reactions; PC-AKI, post-contrast acute kidney injury; eGFR, estimated glomerular filtration rate.

**Remarks:** (i) “Type A reaction” is predictable, common and physiological. It is related to the pharmacological properties of the ICM. (ⅱ) “Type B reaction” is referred to as allergy-like/ hypersensitive reactions. It is unpredictable, uncommon, and is independent of the pharmacological properties of the ICM. (ⅲ) In clinical practice, it is sometimes difficult to differentiate "Type A reaction" from "Type B reaction". To settle this issue, such situation was classified as "uncertain". (ⅳ) “Others” refers to routine examination patients that do not carry the risk items listed above. Given that routine examination patients may also develop ADR, normative risk assessment is also required.

**Supplementary Table 3**. Stratified intervention strategies in the SAW group

| Processing module | Intervention strategy | | High risk | Low risk | No risk |
| --- | --- | --- | --- | --- | --- |
| Clinical communication | Communicate with the clinicians | | √ | √ |  |
|  | The degree of risk factors | | √ |  |  |
|  | Whether examinations are necessary | | √ |  |  |
|  | Whether preventive treatments are needed | | √ | √ |  |
|  | Whether the accompany of the clinician is needed | | √ |  |  |
| Patient communication | Communicate with the patients and their families | | √ | √ | √ |
|  | Communicate with the patients’ authorizers | | √ | √ | √ |
|  | Sign the general informed consent | |  | √ | √ |
|  | Sign the informed consent form with caution | | √ |  |  |
| Whole-process Predictive Process | The sign of risk degrees | | √ | √ |  |
|  | Emergency equipment is at hand | | √ | √ | √ |
|  | The emergency response plan is prepared | | √ | √ | √ |
|  | Electrocardiograph monitoring | | √ |  |  |
|  | Control the dosages of ICM during injection | | √ | √ |  |
|  | Control the speeds of ICM during injection | | √ | √ |  |
|  | Hydration method | Oral hydration | √ | √ | √ |
|  |  | Vein hydration | √ |  |  |
|  | Observation and follow-up | Routine observation |  | √ | √ |
|  |  | Special observation | √ |  |  |

**Abbreviations:** SAW, stratified assessment and warning; ICM, iodinated contrast media.

Note: In principle, enhanced CT examinations were not arranged for high-risk patients. However, the risk-to-benefit ratio should be considered for those who did need examinations due to their clinical conditions.

**Supplementary Table 4.** Comparisons of conventional assessment and SAW regimen

|  | **Conventional assessment period** | **SAW period** | **Notes** |
| --- | --- | --- | --- |
| **Identification of risk factors** | ACR and ESUR guidelines | ACR and ESUR guidelines | Same |
| **Who diagnoses AAR** | Radiologists | Radiologists | Same |
| **Identification of AAR severities** | ACR and ESUR guidelines | ACR and ESUR guidelines | Same |
| **ADR record form** | Yes (**Table S5**) | Yes (**Table S5**) | Same |
| **Initial risk assessment** | By clinicians when ordering examinations | By clinicians when ordering examinations | Same |
| **Risk re-assessment staff** | By radiology nurses with over 8 years of work experience | By radiology nurses with over 8 years of work experience | Same |
| **Risk re-assessment time** | Just before examination | As early as possible post-appointment | Different |
| **Risk re-assessment form** | Conventional assessment form (**Table S2**) | Stratified assessment form (**Table S3**) | Different |
| **Informed consent** | All patients signed a general informed consent form | Ordinary patients signed a general informed consent form. Patients with risk factors signed an informed consent form with caution. | Different |
| **Risk levels** | No stratification | Stratification: high risk, low risk, and no risk (including unknown risk) | Different |
| **Clinical communication** | Just before examination，countermand the examination directly or reschedule for elective examinations | Inform the risk levels in advance, remind the clinic for specific interventions to ensure appropriate and adequate preparation prior to examination | Different |
| **Hydration** | Yes | Yes | Same |
| **Preventive treatments** | Patients with histories of ICM-ADR and other allergies | Patients with histories of severe ICM-ADR, unstable asthma, and severe cardiovascular diseases | Different |
| **Switch to another non-culprit ICM** | No | Yes | Different |
| **ADR record card** | No | Yes | Different |
| **Risk warning label** | No | Yes | Different |
| **Personalized examination programs for high-risk patients** | No | Yes | Different |
| **Observation after examination** | All patients were routinely followed up after examination | High-risk patients were followed up after examination by dedicated staff. | Different |

**Abbreviations:** SAW, stratified assessment and warning; ACR, American College of Radiology; ESUR, European Society of Genitourinary Radiology; ADR, adverse drug reactions; AAR, acute adverse reactions; ICM, iodinated contrast media

**Supplementary Table 5**. ICM-ADR Record Form

| **B1. Patient name: B2. ID number:**  **B3. Hospitalization number: B4. Date of examination: year month day** | | | | | | | | |
| --- | --- | --- | --- | --- | --- | --- | --- | --- |
| gender | 1. male □ 2. female □ | | body weight: | | | age: | | |
| patient source | 1. outpatient □ 2. emergency □ 3. inpatient □ 4. physical examination □ | | | | | | | |
| risk factors |  | | | | | | | |
| iodinated contrast media | 1. Iopromide 370 □ | | 3. Ioversol 320 □ | | | 5. Iopamidol 370 □ | | |
|  | 2. Iodixanol 270 □ | | 4. Iohexol 350 □ | | | 6. Iobitridol 350 □ | | |
| part of examination | 1. head □ 2. neck □ 3. chest □ 4. abdomen □ 5. head and neck CTA □  6. head CTP □ 7. coronary CTA □ 8. limbs □ 9. aorta CTA □ 10. others □ | | | | | | | |
| injection speed: mL/s | | | injection dosage: mL | | | | | |
| **Observation records of adverse reactions** | | | | | | | | |
| occurrence time | 1. at injection min 2. after injection min | | | | | | | |
| symptoms | 1. fever | 2. nausea | | 3. vomiting | 4. itching | | 5. urticaria | 6. excitement |
|  | 7. vascular pain | 8. hoarseness | | 9. sneezing | 10. cough | | 11. chest pain | 12. abdominal pain |
|  | 13. palpitation | 14. facial edema | | 15.chills/shivering | 16. dyspnea | | 17. sudden drop in blood pressure | 18. cardiac arrest |
|  | 19. loss of consciousness | 20. flushing | | 21. other symptoms: | | | | |
|  | * Nausea, fever and other subjective symptoms should be recorded only when the patient complains | | | | | | | |
| treatment method | 1. no need for treatment □ 2. only need to be treated in the examination room □  3. treatment in the emergency room □ 4. require intubation □ 5. require hospitalization □ | | | | | | | |
| severity | 1. mild □ 2. moderate □ 3. severe □ | | | | | | | |
| vital signs | P： R： BP： SPO_2_: | | | | | | | |
| patients whereabouts | 1. emergency room □ 2. inpatient department □ 3. at home □ | | | | | | | |
| treatment method (route and time of administration) | 1. hydration: 1.oral □ 2. intravenous □ | | | | | | | |
|  | 2. oxygen inhalation: 1. nasal catheter oxygen delivery □ 2. oxygen mask □  3. respirator oxygen delivery □ 4. tracheal intubation □ | | | | | | | |
|  | 3. H_1_ receptor blocker: 1. phenanthrene □ 2. diphenhydramine □ | | | | | | | |
|  | 4. dexamethasone: | | | | | | | |
|  | 5. adrenaline: | | | | | | | |
|  | 6. other treatment methods: | | | | | | | |
| outcomes | 1. remission time: 2. symptoms persist: 3. healing 4. death 5. unknown | | | | | | | |
| culprit ICM brands and lot number: | | | | | | | | |

**References**

1 Li X, Chen J, Zhang L et al (2015) Clinical observation of the adverse drug reactions caused by non-ionic iodinated contrast media: results from 109,255 cases who underwent enhanced CT examination in Chongqing, China. Br J Radiol 88:20140491

2 Li X, Liu H, Zhao L et al (2017) Clinical observation of adverse drug reactions to non-ionic iodinated contrast media in population with underlying diseases and risk factors. Br J Radiol 90:20160729

3 Li X, Liu H, Zhao L et al (2018) The effect of preparative solid food status on the occurrence of nausea, vomiting and aspiration symptoms in enhanced CT examination: prospective observational study. Br J Radiol 91:20180198

4 Liu H, Zhao L, Liu J et al (2022) Change the preprocedural fasting policy for contrast-enhanced CT: results of 127,200 cases. Insights Imaging 13:29

5 American College of Radiology Committee on Drugs and Contrast Media. ACR manual on contrast media. Version 10.3. 2018. Available at <https://www.acr.org/Clinical-Resources/Contrast-Manual>. .

6 European Society of Urogenital Radiology. ESUR Guidelines on Contrast Agents. Version 10.0. 2018. Available at <http://www.esur.org/fileadmin/content/2019/ESUR_Guidelines_10.0_Final_Version.pdf>.
